# Back to Basics: Integrating Clinical and Scientific Knowledge to Advance the Field of Trauma—Highlights of the ISTSS-2015

**DOI:** 10.3402/ejpt.v7.33765

**Published:** 2016-11-08

**Authors:** Bernet Elzinga, Christian Schmahl, Miranda Olff

**Affiliations:** 1 Institute of Psychology, Section Clinical Psychology, Leiden University, Leiden, The Netherlands; 2 Department of Psychosomatic Medicine and Psychotherapy, Central Institute of Mental Health, Medical Faculty Mannheim/Heidelberg University, Mannheim, Germany; 3 Department of Psychiatry, Academic Medical Center, University of Amsterdam, Amsterdam, The Netherlands; 4 Arq Psychotrauma Expert Group, Diemen, The Netherlands

The 31st Annual Meeting of the International Society for Traumatic Stress Studies (ISTSS), November 5–7, 2015, was a vibrant and stimulating conference, with many highlights from the opening on the 10th anniversary of Hurricane Katrina; the keynotes of Anke Ehlers, John Krystal, and Regina Sullivan; the vivid panel discussions with recognized leaders in the field of traumatic stress studies; inspiring and eloquent presentations by master methodologists and clinicians; and much more. The atmosphere was excellent, not only within the conference venue, but also outside—we were in The Big Easy. For those who missed it, and for those who like to refresh their memories, we have assembled here in this special issue the highlights of the ISTSS-2015!

The topic of the meeting was “Back to Basics: Integrating Clinical and Scientific Knowledge to Advance the Field of Trauma.” Basic science in the field of stress and trauma has always been an important platform for the development of evidence-based treatments for posttraumatic stress disorder (PTSD). In the past 20 years, research on the effects of trauma and processes of recovery has evolved at great pace. We also know that, despite these advances, the vast majority of those affected by traumatic stress still do not receive optimal care, and even when they do, many patients are not cured after treatment and remain deeply affected by their experiences (Bradley, Greene, Russ, Dutra, & Westen, 2005; Schnyder et al., 2015).

This meeting was a great occasion to reflect on what we have learned so far. The keynote speakers gave excellent overviews on three key research domains in the field of trauma and PTSD. John Krystal took a historical perspective and highlighted advances from 25 years of research, providing an impressive overview on validated pharmacotherapy options for PTSD and ways that the neurobiology of PTSD informs the development of new therapeutic strategies. A review paper on this topic (Kelmendi et al., 2016) can be found in this special issue. Anke Ehlers gave an eloquent overview of the research she and her colleagues conducted to empirically validate and develop evidence-based trauma-focused Cognitive Behavioral Therapy (CBT). She described how systematic observations of re-experiencing in PTSD led to hypotheses about possible cognitive mechanisms that may explain core characteristics of these symptoms and their persistence, and how trauma-focused CBT addresses these mechanisms. In her contribution in this special issue, Anke Ehlers and colleagues describe a promising series of pilot studies on Internet-delivered versions of cognitive therapy for PTSD (Wild et al., 2016). The third keynote speaker, Regina Sullivan, treated us with a comprehensive overview of her fascinating work in rodents on the regulation of the infant brain by a caretaker to alter behavior and facilitate attachment to the caregiver. Readers interested in attachment can enjoy a great overview of her work (Opendak & Sullivan, 2016) in this special issue.

When looking back, we should not only contemplate what we have achieved. It is equally important to consider whether we are changing our views enough as we gather new information about the consequences of trauma exposure and its treatments. In theory, the process of science should ideally result in a continuous evolution of our ideas about trauma and PTSD. In practice, however, it is tempting to view new data through the lens of existing paradigms and interpret findings as supportive of established theories and treatments. These topics were addressed in a vibrant and stimulating panel discussion “What I have Changed My Mind About and Why,” chaired by Rachel Yehuda****. She invited five courageous, outstanding, and highly influential clinician-scholars: David Spiegel, Steven Southwick, Lori Davis, Thomas Neylan, and John Krystal. In this special issue, a summary is provided of the salient points made by each expert and the questions and discussion that ensued as a response to their exciting disclosures (Yehuda et al., 2016). An audio recording of this exciting session can be found here http://www.ejpt.net/public/journals/17/multimedia/132_Invited_Panel.mp3.

Trauma is a global issue (Schnyder, 2013b), and our traumatized patients come from all over the world (Hall & Olff, 2016; Purgato & Olff, 2015). Thus, being sensitive to cultural issues has become a sine qua non for offering good therapy. An invited panel on Culture-Sensitive Psychotraumatology, chaired by Ulrich Schnyder, with Anke Ehlers, Edna B. Foa, Aram Hasan, Gladys Mwiti, and Christian H. Kristensen triggered several important and fundamental questions regarding the treatment of PTSD across the globe. A summary of this panel and additional comments on this topic by leading experts, Frank Neuner, Misari Oe, William Yule, and Richard Bryant, can be found in this special issue (Schnyder et al., 2016).

A third invited panel, chaired by Ruth Lanius, was organized to stimulate the discussion on the social consequences of trauma exposure, focusing on deficits in affect recognition, theory of mind, and empathy in individuals with PTSD. Candice Monson, Andrea Gonzalez, and Margaret McKinnon discussed the relevance to altered social functioning and the intergenerational transmission of trauma and PTSD. A paper on the moral reasoning in women with PTSD related to childhood abuse has been included in this issue (Nazarov et al., 2016).

In this era of exciting innovations, scientists have also made significant progress in several new research domains that are highly relevant to the field of trauma and PTSD. If successfully translated to the clinical realm, these innovative methods may greatly advance our understanding of the effects of trauma and early life. Master methodologist Gustavo Turecki presented a scholarly overview on the methodological considerations when investigating epigenetic consequences of early life adversity, of which a summary can be found in this issue (Fiori & Turecki, 2016). Martin Bohus led a very inspiring invited master clinician session on how to treat concurrent PTSD in patients with borderline personality disorder using dialectical behavior therapy for PTSD. A paper on how state dissociation moderates responses to dialectical behavior therapy for posttraumatic stress disorder from his group is also included in this issue (Kleindienst et al., 2016).

The European Society for Traumatic Stress Studies (ESTSS) and their international partner ISTSS have a long-standing collaboration (Gersons, 2013; Schnyder, 2013a) and we are fortunate to have the possibility to publish important content of meetings of both societies in the *European Journal of Psychotraumatology* (e.g., Dyb & Olff, 2014; Nugent, Sumner, & Amstadter, 2014; Olff & Tan, 2014; Sijbrandij & Olff, 2016). The open access nature of the journal thus opens up the scientific discourse to a wider audience of clinicians, researchers, policy makers, teachers, etc. who were not able to attend the meetings.

With this special issue that presents highlights of the ISTSS-2015, we hope to boost future critical discussions on the advancement of science and evidence-based treatments, and challenge all of you to engage in this process. To optimally advance the field of trauma, we need clever scientists and dedicated clinicians. However, to stay open-minded in this world of constant change and dominant ideologies, we also need “disrupters, disbelievers, sceptics, and revolutionaries” (see Yehuda et al., 2016). Let's welcome them all.

*Bernet Elzinga* Institute of Psychology Section Clinical Psychology Leiden University Leiden, The Netherlands *Christian Schmahl* Department of Psychosomatic Medicine and Psychotherapy, Central Institute of Mental Health Medical Faculty Mannheim/Heidelberg University Mannheim, Germany *Miranda Olff* Department of Psychiatry, Academic Medical Center University of Amsterdam Amsterdam, The Netherlands Arq Psychotrauma Expert Group
Diemen, The Netherlands
